# Effects of a combination of *Poria Cocos*, *Ziziphus spinose*, and gamma‐aminobutyric acid (GABA) on sleep quality and skin health: A randomized double‐blind placebo‐controlled clinical trial

**DOI:** 10.1002/fsn3.4048

**Published:** 2024-02-22

**Authors:** Yining Hao, Weimin Song, Liping Qu

**Affiliations:** ^1^ Yunnan Botanee Bio‐technology Group Co., Ltd. Kunming China; ^2^ YesSkin Medical Aesthetic Clinic Xinya Branch Co., Ltd. Hangzhou China; ^3^ Yunnan Yunke Characteristic Plant Extraction Laboratory Co., Ltd. Kunming China; ^4^ Medaesthee (Shanghai) Biotechnology Co., Ltd. Shanghai China

**Keywords:** integrative medicine, skin health, sleep quality

## Abstract

Sleep is crucial for preserving both physical and mental health, including skin health. Presently, there is a burgeoning interest in the use of herbal and natural ingredients to mitigate the adverse effects of sleep disorders. In this 4‐week, randomized, double‐blind, controlled trial, 70 subjects with sleep disorders were randomly assigned to receive either a placebo or a *Poria cocos*, *Ziziphus spinose*, and GABA (PZG) supplement (10 mL per day). Total sleep duration was detected by wrist actigraphy, and sleep quality was assessed by the Pittsburgh Sleep Quality Index (PSQI). Skin conditions were evaluated based on assessments of skin hydration, glossiness elasticity, color, severity of wrinkles, and skin roughness. After 4 weeks, the total sleep duration significantly increased by 12.96% (*p =* .006) and the PSQI score notably decreased by 59.94% (*p =* .000) compared to the baseline. Notably, compared to the baseline conditions, skin hydration, radiance, elasticity, firmness, wrinkle severity, and roughness were significantly improved in the PZG group. In addition, the PZG group demonstrated significantly greater improvements than the placebo group in terms of changes from baseline in total sleep duration, PSQI score, skin hydration, wrinkle severity, and skin roughness. The present results demonstrated that the combined intake of herbs and GABA can improve sleep quality and enhance skin health without adverse effects.

## Graphical abstract

Poor sleep quality adversely affects skin health. The combination of *Poria cocos*, *Ziziphus spinose*, and GABA improved sleep quality, increased skin hydration, and reduced skin roughness.

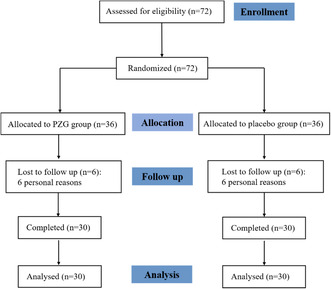

## 1 INTRODUCTION

Insomnia is defined as a subjective dissatisfaction with sleep duration and quality, leading to impaired daytime social functioning (Zhou et al., 2017). It is characterized by difficulties in initiating sleep, maintaining sleep, or waking up early in the morning unable to return to sleep. Chronic insomnia not only impairs cognitive function (Donlea, 2019) but also leads to dysfunction of the immune, circulatory, metabolic, and gastrointestinal systems (Ali et al., 2013; Irwin, 2019; McAlpine et al., 2019; Tobaldini et al., 2019), which can trigger a variety of diseases, such as cardiovascular disease, obesity, hypertension, depression, and severe sleep deprivation, and can even lead to death, significantly affecting people's quality of life. Moreover, chronic insomnia can exacerbate or induce several skin diseases, such as psoriasis and atopic dermatitis (Duan et al., 2021; Hu et al., 2022; Thorburn & Riha, 2010). Previous studies have shown that sleep deprivation or poor‐quality sleep has negative effects on the condition of the skin, including reduced skin moisture, compromised skin immunity, increased skin inflammation, and impaired skin barrier function. Consequently, these factors contribute to accelerated skin aging and pigmentation (Oyetakin‐White et al., 2015).

Insomnia has been linked to dysfunctions in gamma‐aminobutyric acid (GABA) receptor subtypes, specifically the GABA type A (GABAA) and GABA type B (GABAB) receptors (Filip et al., 2006). At present, benzodiazepine receptor agonists are commonly prescribed for insomnia treatment and can enhance inhibitory signals in cell populations that regulate arousal by increasing GABA binding to GABAA receptors. However, the side effects associated with benzodiazepine receptor agonist hypnotics include dizziness, excessive sedation, and even addiction. Additionally, abrupt discontinuation may cause hypnotic‐withdrawal insomnia (Neubauer et al., 2018; Wang et al., 2020). Given the side effects of hypnotic medications and the growing consumer preference for nonpharmaceutical options, it is very important to provide natural and safe nonpharmaceutical alternatives that can enhance sleep quality.

*Poria cocos* (Fuling) is the dried sclerotium of a saprophytic fungus that generally grows on the roots of pine trees. Polysaccharides and triterpenes are the main active ingredients of *Poria cocos* (Rios, 2011). A recent study revealed that oral administration of *Poria cocos* extract can enhance the quality and structure of sleep by reducing sleep latency and increasing the duration of nonrapid eye movement (NREM) sleep in rats with insomnia (Kim et al., 2022). *Ziziphus spinose* (Suanzaoren) is another traditional Chinese herbal medicine commonly used to treat insomnia and anxiety. Saponins, flavonoids, and alkaloids are the main pharmacologically bioactive components in Suanzaoren (Han et al., 2009). Specifically, the triterpene saponins jujuboside A and jujuboside B have been found to have sedative and anxiolytic effects by regulating multiple pathways (Du et al., 2014; Tabassum et al., 2019). Gamma‐aminobutyric acid, widely found in animals, plants, and fermented foods, is a nonproteinogenic amino acid that plays a crucial role in the regulation of neuronal excitability in the central nervous system and acts as an inhibitory neurotransmitter that promotes sleep by suppressing wake‐promoting systems (Hepsomali et al., 2020). Previous studies have suggested that oral GABA may have a positive effect on sleep quality (Byun et al., 2018). However, scientific evidence of the benefits of oral GABA intake alone for sleep health is limited.

Recently, *Poria cocos*, *Ziziphus spinose*, and GABA have been frequently used in oral products to improve sleep quality, but clinical data on their beneficial effects on sleep have been limited. Furthermore, the clinical efficacy of the combination of these three natural ingredients has not yet been reported. Consequently, in this randomized double‐blind controlled clinical trial, we investigated the effects of an oral supplement containing *Poria cocos*, *Ziziphus spinose*, and GABA on sleep quality and skin conditions in women with sleep disorders.

## 2 MATERIALS AND METHODS

### 2.1 Study design

This was a single‐center, randomized, double‐blind, placebo‐controlled, 4‐week (28 days) clinical study. The study group consisted of 70 healthy Chinese women aged 25–55 years with a PSQI score ≥7 (poor sleep quality) and self‐reported skin issues, including dryness, roughness, darkness, and lack of elasticity. Participants were randomly divided into either the test group (PZG group) or the placebo control group, with 35 participants in each group. The two groups were balanced in terms of age and total sleep duration. The primary objective of the study was to observe the changes in sleep quality and skin condition of participants after they consumed a PSG drink for 4 weeks. During the test period, the test group was assigned to receive a drink containing the functional ingredients *Poria cocos* and *Ziziphus spinose* extract (4 mL per 10 mL of PZG drink, containing 11.49 mg of total saponins and 3.32 mg of total flavonoids) and GABA (100 mg per 10 mL of the PZG drink), and the placebo group was assigned to receive a placebo without the functional ingredients for 4 weeks. Neither the PZG supplement nor the placebo differed in appearance. To avoid the potential impact of varied skin care products on the study outcomes, all participants were required to use the same basic skin care products, provided by Yunnan Botanee Biotechnology Group Co., Ltd., which contained only moisturizing ingredients.

### 2.2 Ethics statements

The study was carried out following the rules of the Declaration of Helsinki and approved by the Ethics Committee of Shanghai Clinic Research (Shanghai, China) (approval code: SECCR/2022–091‐01) and Clinical Trials.gov (https://www.clinicaltrials.gov): NCT05908903.

### 2.3 Sample size

Based on the central limit theorem, a larger sample size tends to approximate a normal distribution. Empirically, sample means often exhibit a normal distribution when *N* ≥ 30. Thus, a minimum sample size of 30 participants per group was chosen for the study. Considering potential factors such as a 15% loss to follow‐up, exclusion criteria, and safety observations, a final sample size of 70 participants (35 per group) was determined.

### 2.4 Participants

The participants in the research were healthy Chinese women aged 25–55 years with poor sleep quality (PSQI score ≥7), a body mass index (BMI) ranging from 18 to 24 kg/m^2^, and self‐reported skin issues such as dryness, roughness, darkness, and lack of elasticity. The exclusion criteria were as follows: (1) consumed any food or medicine that included *Poria cocos*, *Ziziphus spinose*, or GABA; (2) consumed any functional food or medicine that may affect sleep, such as sleeping pills or sedatives; (3) had cognitive impairment; (4) had major depressive, severe anxiety, or other underlying psychiatric diseases; (5) were preparing for pregnancy or were pregnant or lactating; (6) had alcohol abuse or dependence; (7) had an allergy history; (8) had irregular sleep habits due to work shifts; (9) participated in any clinical trial assessment within 1 month; (10) had been undergoing treatment for asthma or other chronic respiratory conditions; and (11) had a history of skin diseases (such as psoriasis, eczema, psoriasis, skin cancer, etc.). All participants who were enrolled in this study provided informed consent.

### 2.5 Randomization and blinding

After participants were assessed for eligibility, the data statistician assigned a random number using the RANDBETWEEN function in the Excel range of 1–2 for each group of participants. Subsequently, all the subjects were randomly allocated to either group 1 (test group) or group 2 (placebo control group). The researchers and subjects were blinded to the randomization and allocation of the data.

### 2.6 Evaluation of sleep quality

#### 2.6.1 Detection of sleep duration

Wrist actigraphy was used to automatically monitor sleep duration and confirm the information in real time. It analyzes sleep duration by monitoring the heart rate, and it accurately identifies the sleep–wake points. All the subjects wore the wrist actigraphy device every day during the treatment. Sleep duration data (min) were collected from the subjects before the treatment as well as at 2 and 4 weeks after the ingestion of the product.

#### 2.6.2 Pittsburgh sleep quality index

The Pittsburgh Sleep Quality Index, compiled by psychiatrist Dr. Daniel J. Buysse and his colleagues at the University of Pittsburgh in 1989, is an effective tool for assessing the sleep quality of patients (Thongchumnum et al., 2023). The PSQI includes 18 self‐evaluated items, and the results are categorized into seven factors: (1) sleep quality, (2) sleep latency, (3) total sleep time, (4) sleep efficiency, (5) sleep disorders, (6) use of hypnotics, and (7) daytime dysfunction. The total PSQI score is calculated by summing the scores of the seven subdomains and ranges from 0 to 2 L, with higher scores indicating poorer sleep quality. The PSQI was completed by all subjects at the time of enrollment, and the survey was repeated 2 and 4 weeks after the patients consumed PZG or the placebo.

### 2.7 Evaluation of skin conditions

#### 2.7.1 Objective assessments of skin conditions

Subjects were evaluated before and 4 and 8 weeks after intake of the PZG product or placebo. The faces of all participants were washed; then, the patients were equilibrated in a constant temperature and humidity environment for 30 min (the temperature was maintained at 21°C ± 1°C, and the relative humidity was maintained at 50% ± 10%). Skin hydration and glossiness were detected with a corneometer (Courage & Khazaka, Germany) and a glossymeter (Delfin, Finland), respectively. Skin elasticity and firmness were detected with a Cutometer@MPA580 (Courage & Khazaka, Germany), and the skin tone (ITA°) was detected with a colorimeter (Courage & Khazaka, Germany).

#### 2.7.2 Physician clinical evaluation

A clinician scored the severity of the wrinkles (range from 0 to 9) and skin roughness (range from 0 to 4) based on a scale in which higher scores indicated greater severity.

### 2.8 Safety

Data on adverse events were collected from the time of signing the informed consent form until 14 days after the end of the study. Adverse events and serious adverse events included the type, incidence, severity, severity, and relation to feeding and were reported by the investigator in an adverse event table as part of the case report form.

### 2.9 Statistical analysis

A *t*‐test was used to compare the data between groups. The Wilcoxon signed‐rank test was used when the data were not normally distributed. Statistical tests were performed using a two‐sided test, and the *p* value was calculated. *p* ≤ .05 indicated that the difference was statistically significant. The statistical analysis was performed with SPSS 22. The statistical analysis data are presented as the mean ± standard error (SE).

## 3 RESULTS

### 3.1 Sample size statistics

**FIGURE 1:**
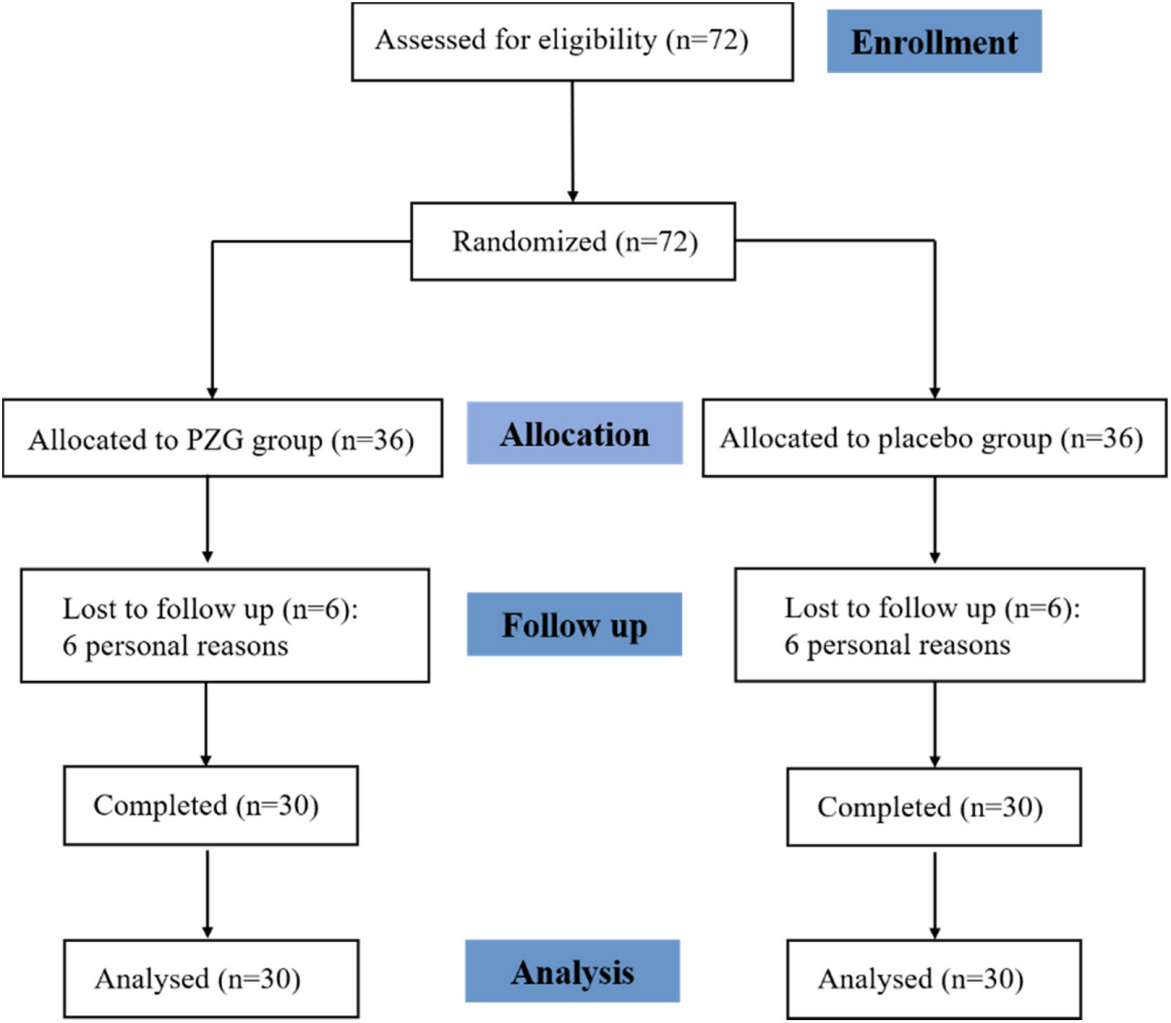
Flowchart depicting the study design.

A total of 72 samples met the eligibility criteria, and 60 samples were analyzed, as shown in Figure 1. According to the statistics, the age of the population was 40.12 ± 7.34 years (the age of the test group was 39.73 ± 7.81 years, and that of the control group was 40.50 ± 6.96 years).

### 3.2 Evaluation of sleep quality

During the testing period, the total sleep time was monitored by wrist actigraphy. As shown in Table 1, the total sleep duration of the PZG group significantly increased after 2 (*p* = .009) and 4 (*p* = .006) weeks compared to the baseline. However, the differences were not statistically significant in the placebo group. The PSQI scores of the PZG group were significantly lower after 2 (*p* = .006) and 4 (*p* = .006) weeks than at baseline. In comparison, the placebo group showed no significant improvement. Moreover, the changes in total sleep duration and PSQI scores from baseline after 4 weeks were significantly greater in the PZG group than in the placebo group (*p* = .000), as shown in Figure 1.

**TABLE 1:** Effects of PZG supplementation on total sleep duration and PSQI scores.

Items	Time (week)	PZG group (*n* = 30)			Placebo group (*n* = 30)			*p* value for change scores (PZG group vs placebo group)
		Mean ± SE	*p* Value (vs. baseline)	Change (vs. baseline)	Mean ± SE	*p* Value (vs. baseline)	Change (vs. baseline)	
Sleep duration	0	390.57 ± 11.02			400.30 ± 13.54			
	2	426.60 ± 11.65	.009	11.73 ± 7.23	405.50 ± 16.10	.894	6.77 ± 8.10	.147
	4	441.20 ± 14.29	.006	12.50 ± 10.54	393.40 ± 19.50	.669	0.40 ± 7.52	.016
PSQI scores	0	10.73 ± 0.44			10.50 ± 0.50			
	2	5.77 ± 0.41	.000	−4.97 ± 0.46	10.23 ± 0.52	.451	−0.27 ± 0.41	.000
	4	4.30 ± 0.44	.000	−6.43 ± 0.55	9.90 ± 0.48	.221	−0.60 ± 0.43	.000

### 3.3 Evaluation of skin conditions

#### 3.3.1 Objective evaluation

**FIGURE 2:**
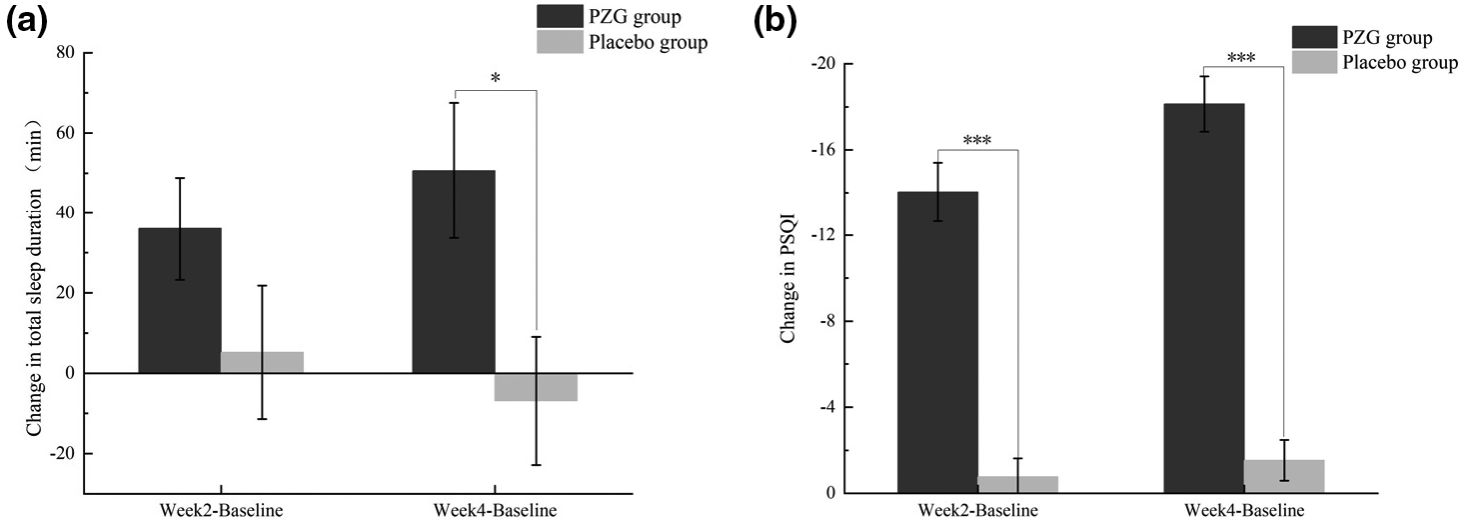
Changes in total sleep duration (a) and PSQI scores (b) (mean ± SE). *p  ≤.05; ***p  ≤ .001 compared to the placebo group.

The changes in skin hydration, skin radiance, skin elasticity, and skin tone ITA° values during the study period are shown in Table 2. Skin hydration was significantly increased in both the PZG group and the placebo group after 4 weeks compared to the baseline condition (*p* = .006). The change in skin hydration from baseline in the PZG group was significantly greater than that in the placebo group (*p* = .001), as shown in Figure 2. After 4 weeks of ingestion, skin glossiness (*p* = .006) and skin elasticity (*p* = .006) in the PZG group were significantly improved, and skin firmness was decreased (*p* = .012) compared with the baseline conditions. However, the changes in skin radiance, skin elasticity (*p* = .606), and skin firmness (*p* = .296) from baseline were not significantly different between the PZG group and the placebo group. In addition, there was no statistically significant difference in skin tone ITA° values between the PZG group and the placebo group (*p* > .05).

**TABLE 2:** Effects of PZG supplementation on skin hydration, radiance, elasticity, firmness, tone, roughness, and severity of wrinkles.

Items	Time (week)	PZG group (*n* = 30)			Placebo group (*n* = 30)			*p* value for change scores (PZG group vs. placebo group)
		Mean ± SE	*p* Value (vs. baseline)	Change (vs. baseline)	Mean ± SE	*p* Value (vs. baseline)	Change (vs. baseline)	
Skin hydration	0	31.57 ± 0.71			32.49 ± 0.82			
	2	37.81 ± 0.96	.000	6.24 ± 0.88	34.60 ± 0.79	.006	2.11 ± 0.71	.001
	4	40.27 ± 1.14	.000	8.70 ± 0.94	36.80 ± 1.24	.000	4.31 ± 1.04	.001
Skin radiance	0	5.66 ± 0.19			5.44 ± 0.16			
	2	6.32 ± 0.15	.000	0.66 ± 0.13	5.81 ± 0.15	.034	0.37 ± 0.15	.258
	4	6.50 ± 0.16	.000	0.84 ± 0.15	6.00 ± 0.13	.000	0.55 ± 0.13	.152
Skin elasticity R2	0	0.57 ± 0.02			0.61 ± 0.02			
	2	0.61 ± 0.01	.165	0.03 ± 0.02	0.60 ± 0.02	.746	−0.01 ± 0.02	.181
	4	0.62 ± 0.02	.032	0.05 ± 0.02	0.64 ± 0.02	.245	0.03 ± 0.03	.606
Skin firmness F4	0	5.88 ± 0.30			5.49 ± 0.23			
	2	5.69 ± 0.26	.503	−0.20 ± 0.29	5.62 ± 0.21	.552	0.13 ± 0.21	.371
	4	5.05 ± 0.26	.012	−0.84 ± 0.31	5.09 ± 0.24	.160	−0.40 ± 0.28	.296
Skin tone ITA°	0	45.94 ± 1.20			47.29 ± 1.19			
	2	44.78 ± 1.38	.082	−1.16 ± 0.76	46.11 ± 1.51	.031	−1.18 ± 0.52	.986
	4	44.74 ± 1.30	.213	−1.20 ± 0.91	45.58 ± 1.56	.022	−1.71 ± 0.70	.906
Skin roughness	0	1.40 ± 0.14			0.90 ± 0.18			
	2	0.80 ± 0.12	.000	−0.60 ± 0.09	1.00 ± 0.19	.257	0.10 ± 0.09	.000
	4	0.57 ± 0.12	.000	−0.83 ± 0.08	1.03 ± 0.16	.346	0.13 ± 0.14	.000
Severity of wrinkles	0	4.17 ± 0.24			4.27 ± 0.31			
	2	3.93 ± 0.20	.161	−0.23 ± 0.15	4.03 ± 0.26	.182	−0.23 ± 0.17	.961
	4	3.40 ± 0.26	.000	−0.77 ± 0.16	4.17 ± 0.25	.467	−0.10 ± 0.14	.003

#### 3.3.2 Physician clinical evaluation

**FIGURE 3:**
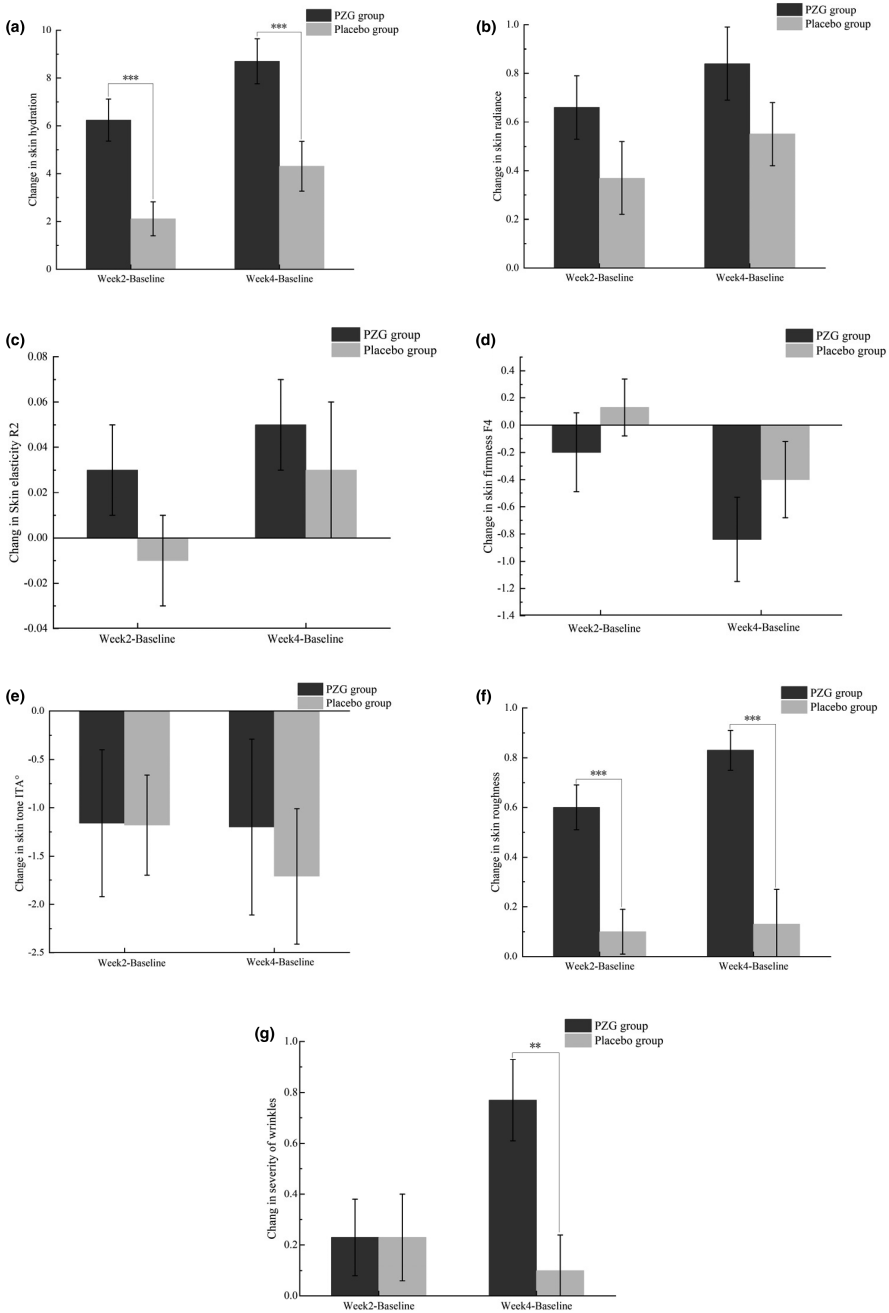
Changes in total skin hydration (a), radiance (b), elasticity (c), firmness (d), tone (e), roughness (f), and severity of wrinkles (g) (mean ± SE). **p ≤ .01; ***p ≤ .001 compared to the placebo group.

Based on physician clinical evaluation data, the PZG group showed a significant reduction in the severity of wrinkles *(p* = .000) and skin roughness (*p* = .000) at 4 weeks compared to the baseline conditions. In addition, the PZG group showed significantly greater changes in wrinkle severity (*p* = .003) and skin roughness (*p* = .000) from baseline than the placebo group, as shown in Figure 3.

### 3.4 Adverse events/serious adverse event records

No adverse events were reported during the study period or within the 14‐day follow‐up period after the study concluded.

## 4 DISCUSSION

Complementary and alternative medicine (CAM) modalities, such as herbal treatments, have gained substantial popularity and are being utilized to prevent and alleviate various health disorders (Hajimonfarednejad et al., 2023; Hashempur et al., 2018; Paudyal et al., 2022), including insomnia (Cooke et al., 2020). While there is much evidence supporting the benefits of traditional Chinese herbal medicines for sleep, clinical studies investigating the reported herbs are limited. In our study, we evaluated the effects of oral supplements containing *Poria cocos*, *Ziziphus spinose*, and GABA on sleep duration, sleep quality, and skin conditions in Chinese females with sleep disorders (PSQI ≥7) and self‐perceived skin issues. The results showed that total sleep duration significantly increased by 12.96% and that PSQI scores notably decreased by 59.94% in the PZG group compared to the baseline values after 4 weeks. Additionally, the changes in total sleep duration and PSQI scores from baseline after 4 weeks in the PZG group were significantly greater than those in the placebo group, indicating that oral PZG supplements can improve the sleep quality of subjects.

*Poria cocos* is reported to exhibit hypnotic effects by enhancing chloride channel activation and GABAergic transmission (Shah et al., 2014). Previous animal studies have provided scientific evidence for the efficacy and pharmacological action of *Poria cocos* as a treatment for insomnia. *Ziziphus spinose* can also exert anxiolytic effects by modulating serotonergic synaptic pathways (Chen et al., 2020). Moreover, Suanzaoren decoction has been shown to improve subjective sleep quality and sleep efficiency, yet PSQI scores remain higher than 5 after 4 weeks of intake (Chan et al., 2015). To date, research on the use of GABA alone to improve sleep quality is very limited. GABA intake may reduce sleep latency through its anxiolytic effects rather than through direct induction or maintenance of sleep. However, when used alone, GABA may not significantly impact overall sleep quality (Cox & Olatunji, 2016; Hepsomali et al., 2020; Maskevich et al., 2020). Notably, the combination of GABA and L‐theanine considerably decreased sleep latency and accelerated sleep duration in mice compared to the effects of the individual use of GABA or L‐theanine. This finding suggests a favorable synergistic effect of GABA/L‐theanine amalgamation on the quality and duration of sleep, in contrast to GABA alone (Kim et al., 2019). Consequently, we hypothesize that the marked increase in total sleep duration and PSQI score observed in our investigation could be attributed to the synergistic effect of *Poria cocos*, *Ziziphus spinose*, and GABA.

Furthermore, the current findings suggest that the consumption of PZG offers advantages not only in terms of sleep well‐being but also in promoting skin health. To minimize any potential impact of skincare products on the outcomes, we ensured that both groups of participants used the same moisturizing skincare products devoid of any other active ingredients. After 4 weeks of supplementation with PZG, the skin hydration level significantly increased by 27.56% compared to the baseline value. Furthermore, the change in skin hydration from baseline in the PZG group was significantly greater than that in the placebo group after 4 weeks. These results suggest that PZG effectively improves the skin water content. The disruption of the hypothalamic–pituitary–adrenal (HPA) axis caused by insomnia leads to increased secretion of glucocorticoids, such as cortisol, which is associated with changes in skin integrity. Increased cortisol levels can reduce hyaluronic acid generation and increase transepidermal water loss (TEWL), which leads to dryness of the skin and impaired skin barrier function (He et al., 2022; Thorburn & Riha, 2010). Conversely, adequate sleep quality can enhance skin barrier function and improve skin hydration.

Based on clinical evaluations by physicians, a notable decrease in the facial severity of wrinkles and skin roughness was observed after 4 weeks of supplementation with PZG. However, no significant differences were found in the control group, indicating that PZG can improve facial wrinkles and reduce skin roughness. It has been reported that poor long‐term sleep quality can diminish skin elasticity and increase wrinkles in women aged 40 (Jang et al., 2020). Therefore, the reduction in skin wrinkles observed in our study may be attributed to the sleep quality‐enhancing effects of the oral PZG supplement. Furthermore, numerous studies have demonstrated the skin‐improving effects of oral antioxidant supplements, suggesting that the observed benefits may also be related to the free radical scavenging activities of *Poria cocos* and *Ziziphus spinose* (Lin et al., 2018; Wu et al., 2004).

Despite significant improvements in skin glossiness, elasticity, and firmness after 4 weeks of PZG ingestion, no significant difference in these skin indices was observed between the PZG group and the placebo group. These findings suggest that the effect of PZG on these skin indices may be attributed to the use of topical skincare products rather than the use of PZG supplementation. In general, clinical studies that observe improvements in skin elasticity and firmness with oral supplementation require longer observation periods, as these improvements are related to the regeneration cycle of collagen in the dermis. A 90‐day clinical study demonstrated that oral ingestion of aloe vera significantly improved wrinkling and elasticity in photoaged skin, increased collagen synthesis, and reduced the expression of the matrix metalloproteinase MMP‐1 (Cho et al., 2009). Regrettably, due to the limited duration of this study, the complete effect of PGZ on skin conditions could not be fully comprehended.

This study presents a novel approach that integrates traditional herbs with contemporary neurotransmitter modulators to enhance sleep quality and skin health. These initial results provide innovative insights for health care professionals and serve as valuable references for the development of more effective interventions. Given the growing popularity of CAM, it is important for health care professionals to be informed about the potential benefits and risks associated with such interventions. However, our study has several limitations. First, our study population was constrained by a relatively small sample size of 70 participants and was restricted to females of a specific age. Additionally, the duration of this study was limited to 4 weeks, preventing a comprehensive understanding of the effectiveness and safety of this treatment. Future studies should extend the study duration, increase the sample size, and expand the population to include males. Furthermore, the inclusion criteria for participants should be further controlled, including socioeconomic status, physical activity, and daily dietary habits.

## 5 CONCLUSIONS

To conclude, oral supplementation with a combination of *Poria cocos*, *Ziziphus spinose*, and *GABA* may have practical application for ameliorating the endogenous factors that impact sleep and skin health. Specifically, our data demonstrated the potential of this oral treatment regimen for improving sleep quality, increasing skin hydration, and reducing skin roughness. However, further research is necessary to thoroughly evaluate the efficacy and safety of this treatment approach, with particular emphasis on increasing the sample size and extending the study duration.

## AUTHOR CONTRIBUTIONS

**Yining Hao:** Conceptualization (equal); data curation (equal); formal analysis (equal); methodology (equal); project administration (equal); software (equal); validation (equal); visualization (equal); writing – original draft (equal). **Weimin Song:** Conceptualization (equal); investigation (equal); methodology (equal); resources (equal). **Liping Qu:** Conceptualization (equal); funding acquisition (equal); investigation (equal); project administration (equal); resources (equal); supervision (equal); writing – review and editing (equal).

## FUNDING INFORMATION

This study was supported by the independent research fund of Yunnan Characteristic Plant Extraction Laboratory, funding code 2022YKZY005.

## CONFLICT OF INTEREST STATEMENT

The authors declare no conflicts of interest.

## INFORMED CONSENT STATEMENT

All participants in the study provided informed consent.

## Data Availability

The data will be made available upon request.
